# Exploring animal enclosures and parasite interactions in Germany

**DOI:** 10.1016/j.ijppaw.2024.101009

**Published:** 2024-10-24

**Authors:** Christopher Sander, Niko Balkenhol, Stephan Neumann

**Affiliations:** a Institute of Veterinary Medicine, Georg-August-University of Goettingen, Goettingen, Germany; b Wildlife Sciences, Faculty of Forest Sciences, University of Goettingen, Goettingen, Germany

**Keywords:** Helminths, Coccidia, Wildlife, Animal enclosures, Investigation, Hotspot-analysis

## Abstract

In order to gain an initial impression of the current parasite situation in wildlife enclosures across Germany, 17 enclosures of six animal species were examined for parasites in soil and fecal samples in seven facilities. Of particular interest in this context are helminths and protists. Despite the potential risks, however, there are only a few studies on parasites in animal enclosures due to the taboo subject. The study examined 661 fecal samples from fallow deer (*Dama dama,* N = 247), wild boar (*Sus scrofa*, N = 207), red deer (*Cervus elaphus,* N = 111), mouflon (*Ovis orientalis musimon* N = 76), roe deer (*Capreolus capreolus*, N = 12) and bison (*Bison bonasus*, N = 8) as well as 136 soil samples from 12 single-species enclosures and 5 mixed-species enclosures. Three hundred and sixty fecal samples (54.46 %) tested positive for parasites using flotation and sedimentation methods. In addition, parasites were detected in 62.5 % (N = 85) of the soil samples. Examination of the faecal samples revealed that the most common parasite species were *Strongyloides* sp., *Trichostrongylus* sp. and *Trichuris* sp. With the help of a Geographic Information System (GIS), the findings could be displayed on an enclosure map and initial hypotheses on environmental relationships could be made. Particularly high parasite samples were mostly located near feeding and resting areas. The results underline the need for more regular monitoring and targeted parasite management to protect the health of the animals. GIS can be used as an additional tool to help identify hotspots and to specifically incorporate the environment into management in order to take animal-friendly measures. This will play a greater role in the future in the context of anthelmintic resistance.

## Highlights

• 661 fecal and 136 soil samples from 17 enclosures were tested across species.
• Seven wildlife parks and six wildlife species in Germany were examined.
• 360 fecal (54.45%) and 85 soil (62.5%) samples were positive for STH and Coccidia.
• GIS can help identify hotspots for targeted parasite management.

## Graphical abstract

**Figure:**
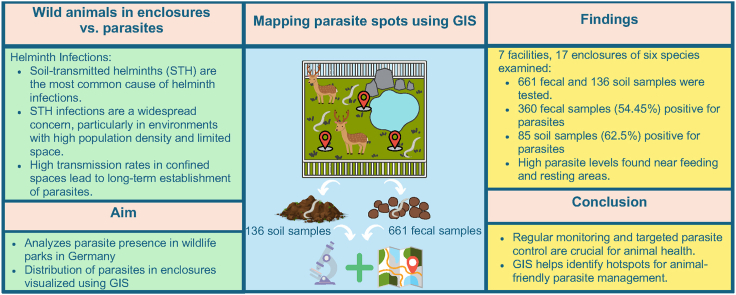

## 1 Introduction

Animal health is becoming increasingly important, especially in the context of risks to humans. In addition to wild animal species, the focus is also increasingly shifting to animals kept in zoological facilities due to their significantly greater proximity to humans. The number of zoos, wildlife parks, zoological gardens, and wildlife enclosures is increasing every year. These facilities play an important role in promoting biodiversity and conservation, research, education, and recreation (Naz et al., 2021; Opara et al., 2010). For example, wildlife parks and zoos house many wild, exotic, and also native species.

Unlike free-living animals, which usually have access to larger areas, animals in enclosures face greater spatial limitations, especially in urban settings where multiple species are often housed together or separately. These restrictions lead to increased stress, loss of genetic diversity, and increased risk of parasitic infections. In particular, endoparasitic infections are a major concern for both free-living and enclosed animals, but the spatial limitations of enclosures facilitate the rapid spread of parasites, allowing them to infect more individuals and establish long-term populations (Fagiolini et al., 2010; Gurler et al., 2010; Kasso and Balakrishnan, 2013).Helminths are of particular interest in this case. These are widely distributed and may cause severe infections. The adult stages live in the gastrointestinal tract, liver and other organs of its hosts. Soil-transmitted Helminths (STH) constitute the most common cause for helminth infections. Some STH are transmitted by eggs that are passed in the feces of infected hosts. Adult worms live in the hosts intestine where they produce thousands of eggs each day. These eggs are deposited in the external environment and contaminate the soil. Animals ingest the eggs and become infected. In contrast, other STH can also enter their host as larvae through the skin. Infected animals can suffer from those parasites a malnutrition, tissue damage and blood loss. Furthermore, this can lead onto chronic and insidious effects on the hosts' health and their nutritional status (Coulson et al., 2018; Stepek et al., 2006).

In addition, some of these parasitic infections represent zoonoses, which allows transmission to humans. This results in risks for animal caretakers as well as visitors. However, despite these known risks, there is little research on this topic (Gurler et al., 2010). The reason for this is that the infestation of wild animals with parasites is sometimes still a taboo subject in these facilities.

This paper examines the situation in wildlife parks and provides a first insight into the current situation of parasites in enclosures across Germany and the management of such facilities. In seven facilities, 17 enclosures of six species were examined for parasites in the soil and fecal samples.

Our study shows high prevalence of various parasites in the investigated enclosures, and suggests that feeding and resting sites within enclosures are hot spots of parasite occurrence. Such knowledge about the occurrence and distribution of parasites can be crucial in the fight against parasite infections.

## 2 Materials and methods

### 2.1 Localities and animals

**Fig. 1:**
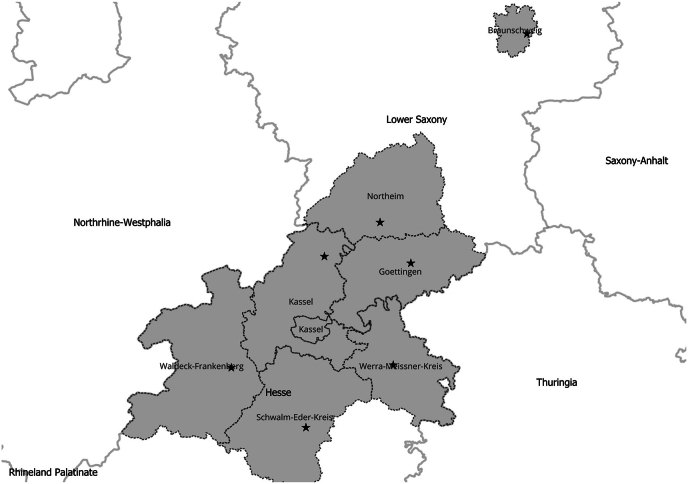
The map of Central Germany shows the location of the seven wildlife parks the sampling took place.

This study was conducted in seven zoological facilities in central Germany in February 2020 to September 2023 (Fig. 1).

The size of the enclosures varied between 0.3 ha and 50 ha. The focus was on wild mammals of the order Artiodactyla (Fallow deer (*Dama dama*), red deer (*Cervus elaphus*), wild boar (*Sus scrofa*), mouflon (*Ovis orientalis musimon*), roe deer (*Capreolus capreolus*) & bison (*Bison bonasus*)). The sampling in each park took place in the morning.

In each enclosure, fresh feces and soil samples were randomly collected at close distances - less than 1 m apart - and their locations recorded using a global positioning system (Garmin eTrex10). The sample size for each enclosure was chosen to cover a variety of areas, e.g. feeding and roosting sites, meadows and forests, as well as high-traffic and low-traffic areas. This ensured a comprehensive representation of the conditions in the enclosures. A total of 17 enclosures (12 single-species enclosures and 5 mixed-species enclosures) and a total sample number of 797 (136 soil samples and 661 fecal samples) were examined. The sample distribution varying greatly between animal species. Most samples came from fallow deer (N = 247) and wild boar (N = 207), while fewer samples were collected from red deer (N = 111), mouflon (N = 76), roe deer (N = 12) and bison (N = 8) (Table 1).

**Table 1:** Overview of the seven wildlife parks by animal species and quantity of fecal samples collected. Fallow deer N = 247, Red deer N = 111, Wild boar N = 207, Roe deer N = 12, Mouflon N = 76 & Bison N = 8.

Parks	Fallow deer	Red deer	Wild boar	Roe deer	Mouflon	Bison
Park 1	26	0	0	0	0	0
Park 2	7	15	14	0	0	8
Park 3	8	10	6	6	6	0
Park 4	88	0	83	0	0	0
Park 5	108	60	88	0	66	0
Park 6	10	10	6	6	4	0
Park 7	0	16	10	0	0	0

Total *N*	247	111	207	12	76	8

### 2.2 Soil sample analysis

For soil analysis, approximately 150 g of soil (5–10 cm depth) was collected from each site with a shovel and placed in labeled polyethylene bags. The location of each sampling site was recorded with a GPS device, along with the corresponding sample number. The soil samples were transported to the laboratory and processed similarly to the spontaneous sedimentation technique described by Rugai et al. (Mandarino-Pereira et al., 2010).

For analysis, 100 g of each soil sample was wrapped in gauze bundles, which were then soaked in a beaker with 125 mL of water at an initial temperature of approximately 45 °C. After 8 days, the bundles were discarded and the supernatants removed. The sediment was collected with a pipette and approximately 90 μL of each sample was placed on a slide with recess (15–18 mm diameter, 0.6–0.8 mm depth) containing approximately 30 μL of Lugol's solution for examination.

The slides were systematically scanned under low magnification (10-20x) in a meandering path from left to right. If a potential parasitic object was detected, the sample was examined more closely at higher magnification (40-60x). The number of helminth eggs/larvae and protists observed was recorded and categorized according to Schmäschke (2014).

### 2.3 Fecal sample analysis

For the fecal analyses, only fresh fecal samples were collected directly from the enclosure ground. The fresh fecal samples were transferred to a plastic bag and also labeled with a unique label. Each fecal sample collection site was also located with the associated sample number using a GPS device. The fecal samples were brought to the laboratory cooled along with the soil samples.

The assignment of the fecal samples to the respective animal species does not present any great difficulties. In the mixed-species enclosures, fecal samples were usually easily identified by size and shape. Red deer feces were usually found as larger and lumpy feces, while roe deer feces were smaller than fallow deer feces. In addition, a gamekeeper was always present in the enclosures to provide support.

Soil-transmitted helminth eggs/larvae and protists were isolated using flotation and sedimentation techniques. The flotation method was used with a focus on the protists and egg stages and the sedimentation method with the previous larval culture was used accordingly for parasitic larvae.

For the flotation technique, approximately 5 g of feces from each sample was mixed with approximately 10 times the volume occupied by the feces. Saturated saline (D at 20 °C: 1.2 g/cm³) was chosen as the flotation medium. A portion of the suspension was passed through a sieve and funnel into a 15 ml centrifuge tube and centrifuged at 1000×*g* for 3 min. Then, about 3–5 drops (about 30 μL) were placed on a slide without recess using a bent platinum wire loop and examined under the microscope. Again, the coverslip was systematically scanned from left to right at low magnification (10-20×) in a meandering fashion, and if there was any indication of a parasitic object, it was enlarged for closer examination at high magnification (40-60x).

In preparation for the sedimentation technique, a larval culture was carried out for 10 days, similar to Dashe and Berhanu (2020). For this purpose, about 10 g of feces were mixed with tap water and crumbled wood pellets in a mortar to form a homogeneous suspension and left to rest for 10 days in a Petri dish. After this time, part of the suspension was passed through a sieve into a funnel and left to rest for 2 h. Then approximately 15 ml was poured into a 50 ml vessel. After standing for 1 h, the water was decanted and refilled. After a further hour, the water was decanted again. Then about 120 μL was removed from the sediment with a pipette and placed on a microscope slide with recess (15–18 mm diameter, 0.6–0.8 mm depth) to be examined under the microscope. As with the other two methods, the coverslip was systematically scanned at low magnification (10-20×) in meandering paths from left to right and, if there was any indication of a parasitic object, it was enlarged in order to examine it more closely at high magnification (40-60x). The number of helminth eggs/larvae and protists observed was again noted and assigned to the sample.

The eggs and protists were identified using the book Koprologische Diagnostik von Endoparasiten in der Veterinärmedizin by Schmäschke (2014). The larvae were identified using the article by Van Wyk and Mayhew (2013). Furthermore, identification charts of the laboratory were used. In addition to the identification of parasites, the intensity of parasite abundance was assessed in accordance with the recommendations of Schmäschke (2014).

A distinction to the free-living nematodes was made on the basis of the corresponding descriptions by Van Wyk and Mayhew (2013) and Schmäschke (2014). For example, free-living nematodes have several characteristics that distinguish them from parasitic nematodes (e.g., rhabditiform esophagus with two conspicuous bulbs caudally).

### 2.4 Enclosure analysis and hotspot identification

All examined wildlife enclosures were largely uniform in terms of their basic structure. In each facility, care was taken to provide the animals with sufficient retreat areas, sleeping, and feeding spots to create a naturalistic environment. Additionally, appropriate stocking densities were maintained to prevent stress and negative impacts on the animals' behavior. The soil pH in all enclosures was neutral, ranging between 6 and 7, and the soil composition was consistently loamy, which ensures good water retention and soil fertility. Some enclosures were partially shaded, providing extra comfort for the animals, especially during the summer months. Water features such as ponds or streams were commonly found in enclosures for red deer, fallow deer, and roe deer, whereas wild boar enclosures had water troughs and wallows instead. Only one of the three wild boar enclosures appeared to have a smaller space requirement. Regarding the prevention of parasitic infections, differences were observed between the facilities. In general, animals are monitored by caretakers and regularly checked by veterinarians. While most facilities applied anthelmintic treatments quarterly or biannually, sometimes as a preventive measure, one park, certified as organic, could only administer medications with special approval and justification. Additionally, two parks occasionally used quicklime alongside anthelmintics in the enclosures to target parasite stages in the soil.

The GPS data were transferred from the Garmin eTrex10 into the open-source software QGIS (Quantum-GIS 3.16 Hannover) and integrated into the enclosure maps for spatial presentation and subsequent investigation. In the process, both the soil sample and fecal sample findings were assigned to the corresponding GPS points. OpenStreetMap satellite data from a QGIS plug-in (QuickMapServices) was used for the mapping.

The GPS points were displayed in color using a parasite score, which reflects the level of infestation per sample. Green describes a low infestation (quantity of detected parasite stages <10), yellow a medium infestation (quantity of parasite stages detected 11–20) and red a high infestation (quantity of detected parasite stages >20). If no parasites were found, the point was shown in white. By looking at the maps, possible hotspots could be highlighted. The observed parasite form (eggs, larvae, coccidia) per slide was selected for quantification. The results of the two methods were combined for the fecal sample examination. The “traffic light” classification was slightly optimized and adapted from the book by Schmäschke (2014) (Schmäschke, 2014).

## 3 Results

### 3.1 Analysis of soil samples

One hundred and thirty-six soil samples were collected in the enclosures. Parasites were detected in 85 soil samples (62.5 %). Parasite structures were frequently found in the soil samples from the animal enclosures, with *Strongyloides* sp. and *Trichostrongylus* sp. detected in most of them. Of particular note was the high percentage of *Strongyloides* sp. in the enclosures of Park 2 (100% in bison samples) and Park 4 (72% in fallow deer samples). *Trichostrongylus* sp. was detected most frequently in Park 4 in wild boar (50%). The presence of *Trichuris* sp. was noticeable in soil samples from the deer enclosure in Park 7 (37.5%). Coccidian infections were found equally in soil samples from wild boar enclosures (33.33%) in Park 3 and Park 6. Also, coccidian parasites were also found in the mixed-species enclosure in Park 6 (25%). They were observed to a lesser extend in red deer (12.5%) enclosures in Park 7 (Table 2).

**Table 2:** Overview of the seven wildlife parks by animal enclosure, number of soil samples collected, parasites observed and their positive findings. N = 136.

Parks	Animal enclosure	Number of soil samples	Parasite species	% of positive samples
Park 1	Fallow deer	12	*Strongyloides* sp.	41.60
			*Trichostronglus* sp	41.60
Park 2	Fallow deer + Red deer	5	*Strongyloides* sp.	80.0
			*Trichostronglus* sp	60.0
Park 2	Red deer	4	*Strongyloides* sp.	50.0
			*Trichostronglus* sp	75.0
Park 2	Bison	2	*Strongyloides* sp.	100.0
			*Trichostronglus* sp	50.0
Park 2	Wild boar	4	*Strongyloides* sp.	50.0
			*Trichostronglus* sp	25.0
Park 3	Fallow deer + Red deer + Roe deer + Mouflon	14	*Strongyloides* sp.	50.0
			*Trichostronglus* sp	21.40
			*Coccidia*	21.40
Park 3	Wild boar	3	*Strongyloides* sp.	33.33
			*Coccidia*	33.33
Park 4	Fallow deer	25	*Strongyloides* sp.	72.0
			*Trichostronglus* sp	56.0
			*Trichuris* sp.	0.04
Park 4	Wild boar	16	*Strongyloides* sp.	68.75
			*Trichostronglus* sp	50.0
			*Trichuris* sp.	0.04
Park 5	Fallow deer	7	*Strongyloides* sp.	85.71
			*Trichostronglus* sp	57.14
Park 5	Red deer	9	*Strongyloides* sp.	77.77
			*Trichostronglus* sp	77.77
Park 5	Wild boar	4	*Strongyloides* sp.	50.0
			*Trichostronglus* sp	50.0
Park 5	Mouflon	–	*-*	–
Park 6	Fallow deer + Red deer + Roe deer + Mouflon	14	*Strongyloides* sp.	16.66
			*Trichostronglus* sp	16.66
			*Coccidia*	25.0
Park 6	Wild boar	3	*Strongyloides* sp.	0.0
			*Trichostronglus* sp	0.0
			*Coccidia*	33.33
Park 7	Red deer	8	*Strongyloides* sp.	87.50
			*Trichostronglus* sp	75.0
			*Trichuris* sp.	37.5
			*Coccidia*	12.5
Park 7	Wild boar	6	*Strongyloides* sp.	66.66
			*Trichostronglus* sp	50.0
			*Trichuris* sp.	16.66

### 3.2 Analysis of fecal samples

Three hundred and sixty fecal samples tested positive for parasites using flotation and sedimentation methods. This represents a total percentage of 54.46%. Examination of the fecal samples revealed that the most common parasite species were *Strongyloides* sp., *Trichostrongylus* sp. and *Trichuris* sp. The prevalence varied between species, with wild boar showing a particularly high prevalence of *Trichuris* sp. (15.89%). Fallow deer and red deer showed similar prevalence values for *Strongyloides* sp. (35.1% and 28.78% respectively). Mouflon and bison also showed relevant parasite detections, albeit to a lesser extent. *Nematodirus* sp. was observed particularly frequently in roe deer feces (20%), but was also found in smaller quantities in the other wild animals except for red deer. *Ostertagia* sp., on the other hand, was only found in the feces of red deer, albeit in small quantities (0.72 %). Among the protists, only coccidian parasites were observed. With the exception of roe deer, these were present in all wild animals and were also found particularly frequently. Here, wild boar should be mentioned first (21.96 %)(Table 3).

**Table 3:** Parasite prevalence in fecal samples by animal species. Fallow deer N = 247, Red deer N = 111, Wild boar N = 207, Roe deer N = 12, Mouflon N = 76 & Bison N = 8.

Parasite species	Fallow deer	Red deer	Wild boar	Roe deer	Mouflon	Bison
	*%*	*%*	*%*	*%*	*%*	*%*
*Strongyloides* sp.	35.1	28.78	30.07	60.01	34.97	28.78
*Trichostronglus* sp.	28.98	30.21	29.91	20.0	33.57	27.78
*Trichuris* sp.	14.7	15.83	15.89	0	17.48	16.67
*Nematodirus* sp.	0.82	0	1.17	20.0	2.09	5.56
*Ostertagia* sp.	0	0.72	0	0	0	0
*Coccidia*	20.41	24.47	21.96	0	11.89	22.22

The prevalence of parasites in the fecal samples varied significantly between the wildlife parks. Park 4 showed the highest prevalence values for *Strongyloides* sp. (60.82%) and *Trichostrongylus* sp. (59.65%). In contrast, Park 2 and Park 6 had lower prevalence values, especially for *Strongyloides* sp. (31.81% and 25.0%, respectively). The prevalence of *Trichuris* sp. also varied between parks, with the highest values recorded in Park 4 (39.77%) and Park 5 (27.64%). For *Ostertagia* sp. the data show a moderate to low prevalence, with peaks in Park 7 (19.23%) and Park 3 (11.11%). Higher prevalences were consistently observed for coccidian parasites. Park 2 had the highest prevalence (59.09%) and Park 1 the lowest (23.07%)(Table 4).

**Table 4:** Comparison of parasite prevalence in fecal samples by wildlife park.

Parasite species	Prevalence Park 1 (%)	Prevalence Park 2 (%)	Prevalence Park 3 (%)	Prevalence Park 4 (%)	Prevalence Park 5 (%)	Prevalence Park 6 (%)	Prevalence Park 7 (%)
*Strongyloides* sp.	38.46	31.81	27.78	60.82	54.35	25.0	57.69
*Trichostronglus* sp.	38.46	36.36	27.78	59.65	47.83	22.22	53.85
*Trichuris* sp.	23.07	20.45	19.44	39.77	27.64	13.89	15.38
*Nematodirus* sp.	7.69	6.82	0.00	12.28	5.9	2.78	0.00
*Ostertagia* sp.	0.00	0.00	11.11	6.43	4.04	2.78	19.23
*Coccidia*	23.07	59.09	47.22	43.27	27.02	44.44	26.92

### 3.3 Evaluation of the enclosure maps for hotspot analysis

Parasite observations were quantified using a score system, which was visualized using a traffic light color display. The sample points were evaluated according to the frequency of the parasite forms. For the fecal samples, the parasite score was determined from the results of the flotation method and the sedimentation method. Coccidia, parasite eggs and larvae were recorded together. The same applies to the soil samples. The total number was then compared again with the reference.

The analysis of the samples found in the 17 animal enclosures revealed that the detection of parasite forms was distributed differently within the enclosures. Despite the different distribution patterns, there could be a correlation between the detection of higher parasite forms and certain areas in many enclosures. It appears that feeding and resting areas in particular tended to have higher parasite concentrations.

**Fig. 2:**
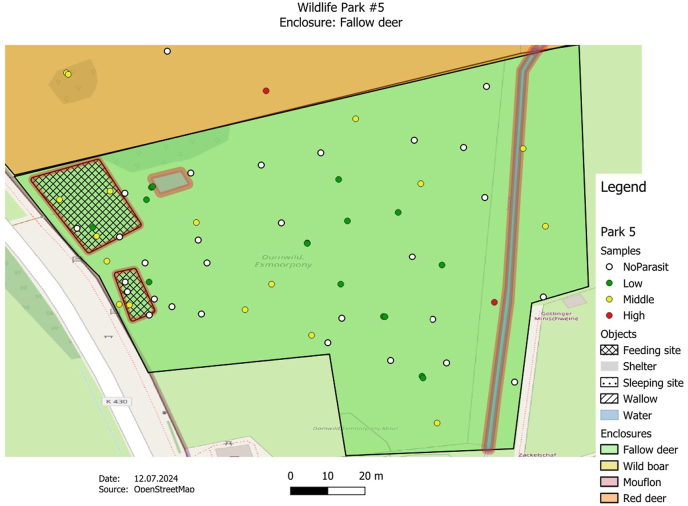
The map shows a fallow deer enclosure (park 5) with the distribution of soil and manure samples. The crossed areas represent the approximate feeding places of the animals. The enclosure is characterized by a grassy landscape with a stream flowing through it. The gray area in the northwest of the enclosure is a shelter for the animals. According to the aerial photo analysis, the enclosure has an approximate area of 1.5 ha. Larger parasite findings were made at the feeding areas and at one place by the stream. Others were found scattered throughout the enclosure.

In the fallow deer enclosure (Fig. 2), for example, a generally low to medium number of parasite forms were found, whereby no parasite forms could be observed in samples from most areas. The positive findings here were concentrated near feeding areas, for example, which indicates possible transmission routes between the animals.

**Fig. 3:**
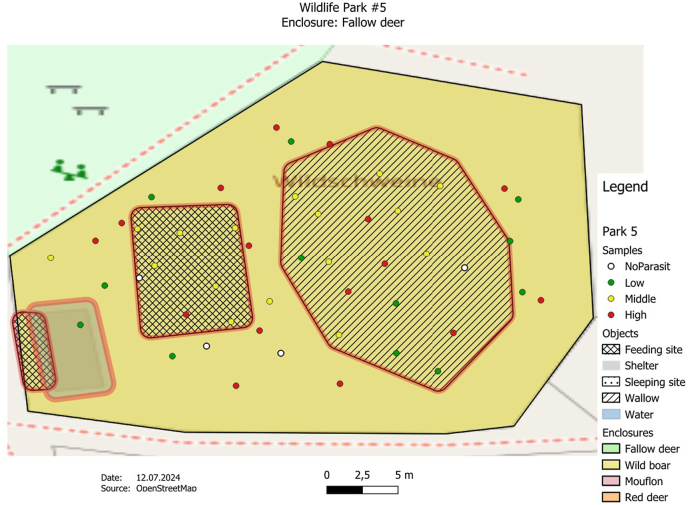
The map shows a wild boar enclosure (park 5) with the distribution of soil and droppings samples. The crossed areas represent the approximate feeding areas for the animals. The enclosure is characterized by a marshy landscape with a large wallow (lined area). According to the aerial photo analysis, the enclosure has an approximate area of 0.2 ha. Due to the moist soil structure of the smaller enclosure, heavy parasite findings were observed over the entire area.

In the example of a wild boar enclosure (Fig. 3), a higher number of parasite forms were observed in soil and fecal samples, particularly in the eastern area of the enclosure. The enclosure also has a larger number of medium to high quantities of observed parasite forms. The positive samples here were scattered over the entire area, which could possibly indicate a greater spread of parasites within the enclosure.

**Fig. 4:**
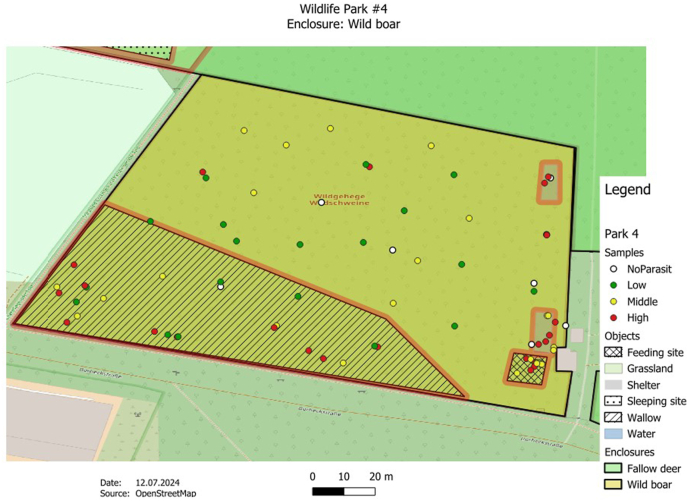
The map also shows a wild boar enclosure (park 4) with the distribution of soil and excrement samples. The crossed area represents the approximate feeding area for the animals, although visitors can also feed the animals outside the wallow area (lined area) in this enclosure. The enclosure is also largely characterized by a marshy landscape. According to the aerial photo analysis, the enclosure has an approximate area of 3 ha. Particularly heavy parasite findings were observed in the area of the wallow, the feeding area and the shelters.

Another wild boar enclosure is shown in Fig. 4. Higher numbers of parasite forms were observed around the wallow, feeding area and shelters. The larger wallow is located directly next to the enclosure fence, where visitors can also feed the animals.

## 4 Discussion

To our knowledge, this study is the first comparative study in German wildlife parks on the prevalence and distribution of soil-transmitted helminths in wild animals kept in enclosures. In addition, most studies primarily focus on zoos. Therefore, these results provide valuable insights into the distribution of parasites in different wildlife parks and species. Parasites in animal enclosures pose significant concerns for animal health and welfare (Barbosa et al., 2020). Understanding the prevalence and distribution of parasites within enclosures is crucial for effective management and mitigation strategies. Parasite form and their distribution in enclosures vary depending on factors such as design, size, environmental conditions and animal density.

The frequent observations of *Strongyloides* sp., *Trichostrongylus* sp., *Trichuris* sp. and coccidia in both fecal and soil samples may indicate widespread environmental contamination. Nematode infections and coccidian infections are the most common parasite infections. This increased observation of nematodes and coccidia is due to their direct life cycle, which allows rapid transmission via contaminated food and soil without the need for intermediate hosts (Ferdous et al., 2023). These results are consistent with previous studies (Dashe and Berhanu, 2020; Ferdous et al., 2023; Gałęcki et al., 2015; Getachew et al., 2017; Goossens et al., 2005; Gurler et al., 2010; Opara et al., 2010; Parsani et al., 2002; Pérez Cordón et al., 2008).

The frequent infections with *Strongyloides* sp. in Artiodactyla reflect the observations in the study by Ferdous et al. (2023) (Ferdous et al., 2023). Similarly common is the observation of *Trichostrongylus* sp. by the ability of strong environmental contamination (Gałęcki et al., 2015). Parsani et al. (2002) describe that coccidian infections are frequently associated with nematode infections, a finding that is also reflected in our results (Parsani et al., 2002). This correlation is also confirmed by the study of Pérez Cordón et al. (2008), in which coccidia showed the highest prevalence in Artiodactyla and were mostly associated with *Nematodirus* sp. infection (Pérez Cordón et al., 2008). This is also shown by our results in relation to *Nematodirus* sp. However, it is interesting to note that no coccidia were observed in roe deer in our study, while *Nematodirus* sp. had the highest prevalence here. However, this could be related to the small sample size (N = 12). Pérez Cordón et al. (2008) also note in their study that fecal samples with coccidia and *Nematodirus* sp. were usually more liquid. This was also shown by the positive bison fecal samples (Pérez Cordón et al., 2008). The observation of high coccidia prevalences in our study is also confirmed in other studies (Fagiolini et al., 2010; Gałęcki et al., 2015; Naz et al., 2021; Pérez Cordón et al., 2008). The reason for the frequently high prevalences is the presence of infectious oocysts in the soil, in vegetation and water bodies (Jolley and Bardsley, 2006). Jolley and Bardsley (2006) also report that despite often high coccidian infection, infected animals show no symptoms. This was also observed in this study. Despite high parasite levels in the feces, many animals showed no clinical signs of infection, as Opara et al. (2010) also found (Opara et al., 2010). This suggests that many wild animals can harbor parasites without showing noticeable symptoms (Opara et al., 2010).

Gurler et al. (2010)notes that *Trichuris* sp. is a common helminth, which is consistent with our findings (Gurler et al., 2010). A similar observation of higher prevalences was also made by Galecki et al. (2015) in his study, both in deer and wild boar (Gałęcki et al., 2015). In this study, only wild boars could be examined as the only omnivores. Nevertheless, other studies confirm the frequent findings of protists, *Trichostrongylus* sp. and *Trichuris* sp. in this species (Gałęcki et al., 2015; Pilarczyk et al., 2024; Silva and Müller, 2013).

*Ostertagia* sp. was only sporadically observed in red deer in this study with an overall prevalence of only 0.72%. To the authors' knowledge, there are only few studies on *Ostertagia* sp. in wild animals in enclosures. Gossens et al. (2005) report higher prevalences of *Ostertagia* sp. in cervids and point out that infective larvae on pastures are the main source of infection (Goossens et al., 2005).

No infections with cestodes or trematodes were detected, which is consistent with other studies (Dashe and Berhanu, 2020; Parsani et al., 2002).

When comparing the prevalence rates between the parks, the high numbers in Park 4 are particularly noteworthy. These increased rates are probably due to the husbandry conditions. As Park 4 is a certified organic park, the use of medication or similar treatments is restricted and requires approval, which also applies to the treatment of the enclosure floors. In Park 5, however, the wild boar enclosure was significantly smaller than in the other parks, covering only about 0.2 ha. This smaller enclosure combined with a large wallow probably favored increased parasite transmission and reinfection rates. There were no other particular differences between the enclosures in all parks.

Environmental conditions, animal density and management practices play an important role in the spread of parasites. Environmental conditions are crucial for the spread of parasites. Abiotic and biotic factors in enclosures can increase the likelihood of successful parasitic infections. Soil-transmitted helminths often find optimal conditions for dispersal and reinfection due to resistant eggs (Barbosa et al., 2020; Panayotova-Pencheva, 2013). In this and other studies, multiple infections and the year-round presence of certain parasite species were frequently observed, indicating continuous transmission and reinfection (Panayotova-Pencheva, 2013).

Wildlife parks offer more natural habitats than zoos, but enclosures still significantly restrict the range of movement of animals compared to their wild relatives. High animal densities in enclosures increase the risk of infection, and parasites can be introduced by new animals via human activities (Panayotova-Pencheva, 2013). Weinstein and Lafferty (2015) describe how human activities affect wild animals and their parasitic nematode infections by promoting infections by creating new links through species introductions and strengthening existing transmission dynamics through higher animal densities, which increases parasite survival and host-parasite contact rates (Weinstein and Lafferty, 2015).

Effective parasite control in animal enclosures requires a multifaceted approach, including regular monitoring, appropriate hygiene practices, environmental management and targeted treatment strategies. Regular fecal analysis is critical to diagnosing infections and reinfections.

Naz et al. (2021) emphasize that the results of faecal analyses are important for the development of treatment and management plans to reduce infections (Naz et al., 2021). Barbosa et al. (2020) emphasize the advantage of sampling. By taking faecal and soil samples, a minimization of animal stress can be observed, in contrast to the use of chemical or mechanical means (Barbosa et al., 2020). The additional examination of soil samples together with fecal samples can provide a more comprehensive picture of parasite distribution in wildlife parks. However, as in Manderino-Pereira's study, there are few studies examining soil samples in animal enclosures as there is no gold standard for parasitic examination (Mandarino-Pereira et al., 2010). Possibly due to the chosen methodology, time period and or conditions for larval culture and soil isolation, fewer parasites were isolated than from the fecal samples.

The mapping of parasite foci in enclosures is of great scientific importance as it enables a better understanding of the distribution and concentration of parasites. This knowledge is crucial to develop targeted parasite control measures, improve wildlife health and minimize zoonotic risks (Hernández et al., 2018; Mascarini-Serra, 2011). GIS-based mapping of sampling points with traffic light colors can help to visually represent the geographical distribution of parasites and identify potential hotspots of parasitic activity. Targeted measures can be taken at these points to reduce the parasite load and thus contribute to animal health. GIS is also ideally suited to integrate spatially referenced data by correlating sample types with geographical locations and enabling GIS-based techniques such as spatial regression and interpolation (Di Lorenzo et al., 2023). Using the information already available on animal husbandry, environmental factors and hygiene measures of park operators, parasite control measures can be further adapted and optimized. In the cartographic analyses of this study, certain areas, such as feeding areas, shaded resting and staging areas and poorly drained areas, are shown to be potentially conducive to parasite survival and reproduction. This is supported by the results of the samples from these specific locations. These observations indicate that targeted measures should be taken in these areas to reduce the parasite load and improve the health of the animals.

Despite the significant results, the results must be interpreted with caution due to limitations. Although the samples were taken as fresh as possible, some may have been lying on the ground for a while, making some stages undetectable. Accurate information on animal density and parasite load was not available due to a lack of information on the size and number of animals in each enclosure. No additional tests such as PCR were performed to confirm the results. Further studies are needed to gain more insight into the parasitic situation of wild animals in enclosures. When detecting *Strongyloides* sp. it is important to note that distinguishing the eggs of *Strongyloides* sp. from other species in the Rhabditidae family can be difficult. The most reliable method is to collect faecal material directly from the rectum. Therefore, it is possible that eggs of non-parasitic species of the Rhabditidae family may have been detected.

There are different diagnostic methods for parasites, each with advantages and disadvantages. This study chose the flotation and sedimentation method (morphological identification) as both eggs and larvae could be identified from the literature. These methods are commonly used and optimal for identification (Broussard, 2003; Verocai et al., 2020). ELISA for identification of nematodes is currently not available. PCR was not chosen due to lack of reference materials and difficulties in standardization (Seesao et al., 2017). Sabatini et al. (2023) point out that currently no technique has a low detection limit with high accuracy and precision. They emphasize the need to be aware of the advantages and limitations of the chosen method.

Overall, this study demonstrates the need for regular surveillance and targeted parasite management strategies in wildlife parks to protect animal health and control the spread of parasites. GIS mapping can provide an additional, useful tool to visually represent potential hotspots and assist zoological facilities in the management of animals. These require comprehensive management and surveillance strategies to minimize infection risks anyway (Getachew et al., 2017; Panayotova-Pencheva, 2013). A deep understanding of the biotic and abiotic factors associated with animal density is crucial for maintaining animal health. GIS mapping can further deepen this understanding and provide valuable insights. Further studies could investigate GIS mapping and the adaptation of management practices to parasite prevalence in order to test more sustainable and effective measures.

## 5 Conclusion

In conclusion, our results show that parasites are quite common in both soil and fecal samples and that the enclosure management could be an important factor for distribution of parasites within enclosures. Analyzing the feces and soil in enclosures and mapping parasites plays a crucial role in understanding the diversity, distribution and dynamics of parasites in enclosures and enables managers to take effective control measures to reduce the risk of infection. By taking a proactive approach to parasite control, enclosures can provide a healthier and safer environment for animals and humans. Further research into novel control strategies and sustainable management practices is critical to minimizing the impact of parasites in animal enclosures.

## CRediT authorship contribution statement

**Christopher Sander:** Writing – original draft, Investigation, Formal analysis. **Niko Balkenhol:** Writing – review & editing, Software. **Stephan Neumann:** Supervision, Project administration, Funding acquisition.

## Ethical standards

Not required.

## Financial support

This project was funded by the Federal Ministry of Economy and Technology (Project ZIM ZF4351502MD9).

## Declaration of competing interest

The authors declare no conflict of interest.
